# Determining the metrics of competence in robotic hysterectomy: a systematic review

**DOI:** 10.1007/s11701-025-02471-6

**Published:** 2025-06-13

**Authors:** Kayla Arcamo, Sita Murugappan, Kirsten Larkins, Helen Mohan, Anthony Costello, Adam Pendlebury, Orla McNally, Rosie McBain

**Affiliations:** 1 International Medical Robotics Academy, Parkville, Melbourne, Australia; 2 University of Melbourne, Parkville, Melbourne, Australia; 3 Peter MacCallum Cancer Centre, Parkville, Melbourne, Australia; 4 The Royal Women’s Hospital, Melbourne, Australia

**Keywords:** Robotic hysterectomy, Robotic surgical education, Metrics of competence, Robotic hysterectomy curriculum

## Abstract

With the rapidly increasing use of robotic-assisted surgery in gynecology, there is a clear need for a structured robotic hysterectomy curriculum. To develop an effective training program, valid performance metrics that reliably assess skill level is required. As part of robotic curriculum development with IMRA using Kern’s framework, this systematic review aims to identify proposed metrics of competence and assess their validity within the context of robotic hysterectomy training. A systematic literature search of OVID MEDLINE and EMBASE was conducted following the PRISMA guidelines, with keywords related to ‘hysterectomy’, ‘robot-assisted’, and ‘metric’. The study aims, methods, outcomes, description of metrics, measurements of metrics, and metrics validity were extracted and analyzed. The initial search yielded 531 articles, of which 3 were included. Three additional articles were identified through citation and website searching, resulting in a total of six articles being included in this review. Development and identification of both simulator and intraoperative metrics greatly varied between the studies. Several studies utilized an expert consensus-based methodology, such as a modified Delphi methodology, to develop performance metrics. All metrics were assessed for content, construct, and predictive validity. Two studies were able to demonstrate predictive validity; however, there was limited correlation between virtual reality simulator metrics and intraoperative scores. This review highlights the lack of standardized and validated metrics specific to robotic hysterectomy, as well as the inability of established assessment tools to differentiate between robotic surgical skill level. This forms the context for ongoing work at IMRA to develop a granular assessment tool to assess skill acquisition as part of a robotic hysterectomy curriculum.

## Introduction

Robotic-assisted surgery is fast becoming the next step in the advancement of minimally invasive gynecologic surgery [1]. Robotic-assisted surgery has the potential to improve technical intraoperative performance of complex gynecologic cases, by overcoming limitations of conventional laparoscopic surgery, e.g., restricted range of motion of the surgical instruments, through high-resolution visualization and improved ergonomics. Robotic surgery has been associated with improved patient outcomes, early recovery, and decreased hospital length of stay [1, 2]. Robotic-assisted laparoscopic hysterectomy (RALH) is currently the most commonly performed application of robotic surgery in gynecology and as the number of robotic cases increases, so too has the interest in robotic training [3]. Trainees face numerous challenges. however, with limited operative exposure, increasing costs and individualized robotic credentialing bodies hindering the number of trainees completing fellowship training with the required proficiency to perform gynecologic robotic surgery [4, 5].

It is becoming increasingly clear that a structured and objective robotic hysterectomy curriculum is required to address the increasing uptake of robotic surgery. For a training curriculum to be effective, metrics that are procedure-specific, objective, and reliably assess skill progress are required [6, 7]. A metric- or competence-based educational pathway ensures that the learners reach the knowledge, skills, and attitudes to a desired level, before progressing forwards [8]. Compared to a time- or case-based curriculum, a metric-based system has been shown to improve trainee performance and reduce intraoperative complications [9].

Validated metrics are an important component of a training program. Kern et al. developed a six-step approach to curriculum development, whereby the steps “Goals And Objectives and Evaluation And Feedback” serves to identify specific and measurable objectives and opportunities for formative feedback for the individual learner and ensuring the curriculum is meeting its goals [10, 11]. While neither have been developed specifically for robotic hysterectomy, several tools have been created that assess surgical performance, such as the Global Operative Assessment of Laparoscopic Skills (GOALS) and the adapted version for robotics Global Evaluative Assessment of Robotic Skills (GEARS). GEARS has been validated to measure robotic surgical proficiency but is not procedure-specific and relies on subjective assessor feedback, potentially introducing an element of bias [12, 13].

Collectively, there is a need for the development and validation of performance metrics that allow a structured way for learners to evaluate their performance and reach a proficiency benchmark. During the development of a robotic curriculum at IMRA for hysterectomy, this review was conducted to inform the curriculum design within Kern’s framework by evaluating what the existing knowledge base is regarding performance metrics in this setting. This study, therefore, will address the “Goals And Objectives and Evaluation And Feedback” sections of Kern’s curriculum development method for a robotic hysterectomy curriculum at IMRA [11]. This review aims to identify the metrics of competence in assessing robotic hysterectomy performance and assess their validity in measuring surgical skill level.

## Methods

**Fig. 1:**
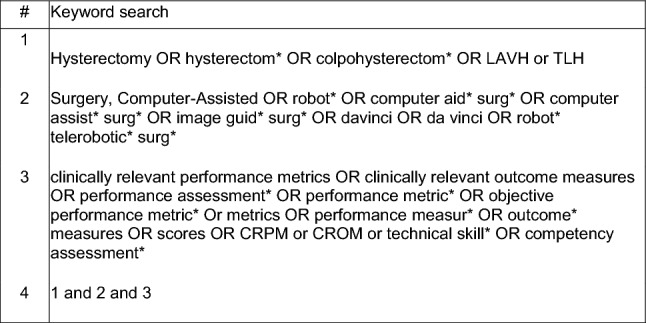
Keyword strategy with combined Boolean operators

In this systematic review, electronic databases OVID MEDLINE and EMBASE were searched for primary studies that described metrics of competence in robotic hysterectomy surgery and assessed their validity in robotic gynecology training. A systematic search was conducted in May 2023 following the PRISMA guidelines. Key search terms and Boolean operators are described in Fig. 1. Additional articles were obtained via citation searching of included articles as well as searching the websites of known organizations involved in robotic hysterectomy training.

### Inclusion and exclusion criteria

All relevant articles published prior to the search date were included. Only full-text articles written in English were included. The studies that were included identified metrics to assess robotic hysterectomy performance and addressed the validity and reliability of these metrics. Studies that did not clearly describe objective metrics were excluded, as were articles that proposed performance metrics without taking measures to assess their validity. All settings of soft-tissue robot-assisted surgeries, dry laboratory, wet laboratory, animal models, and in vivo operating were included. Studies addressing laparoscopic surgery or not addressing robotic hysterectomy surgery specifically were excluded. Forms of study design including conference abstracts, committee opinions, and research letters were also excluded.

### Data extraction

For the included articles, data were extracted using a predetermined list including authors, study aims, description of methods, participant characteristics, outcomes, description of metrics, measurements of metrics and metric validity.

## Results

**Fig. 2:**
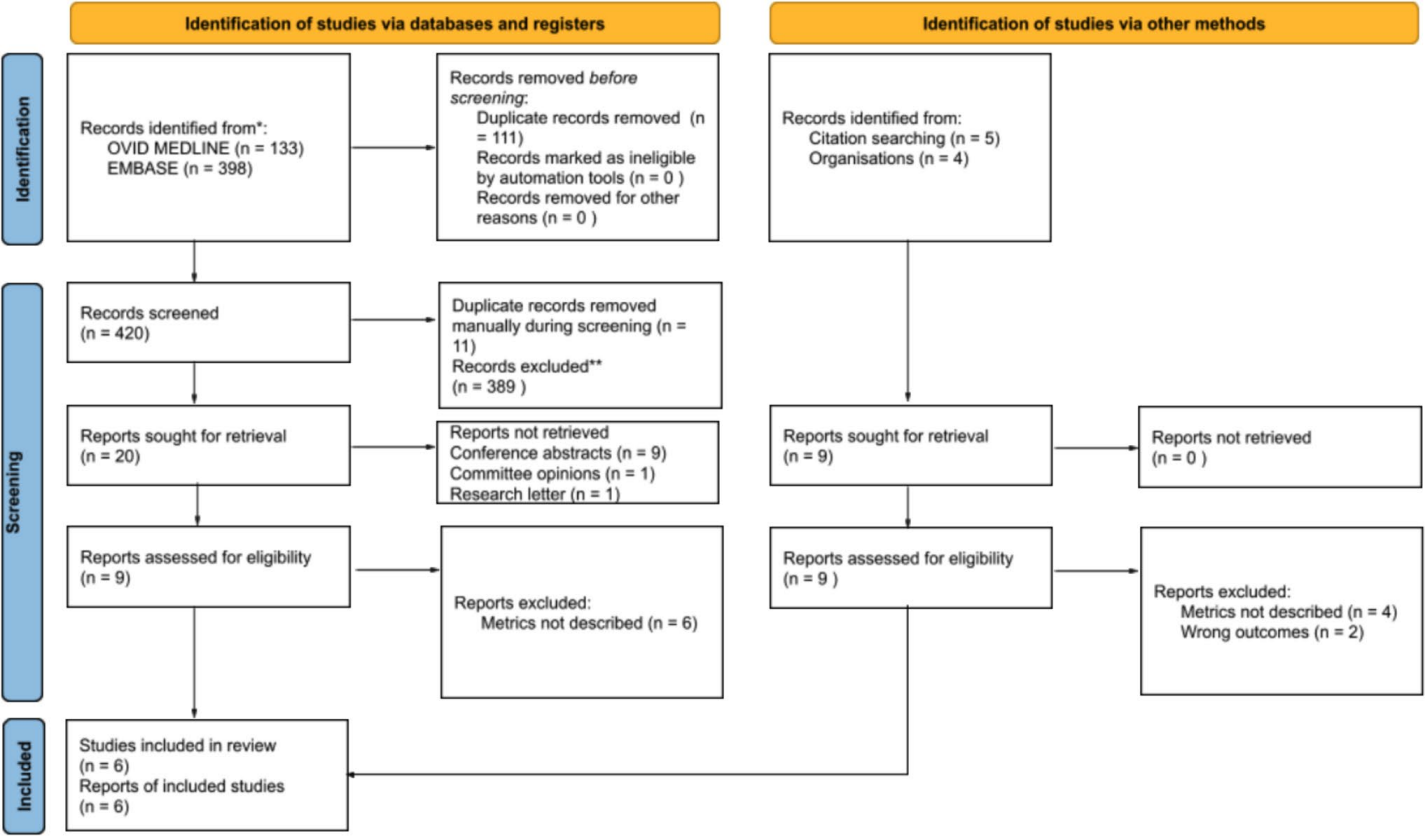
PRISMA diagram of the systematic search strategy

The primary database search yielded 531 articles—133 from Ovid Medline and 398 from EMBASE. Following removal of duplicates before screening and manual removal during title and abstract screening, 409 unique articles remained. Twenty articles were selected for full-text review following title and abstract screening. Eleven articles were not retrieved due to being conference abstracts (*n* = 9), committee opinions (*n* = 1), and research letters (*n* = 1). From the remaining nine articles, six were excluded for not describing performance metrics. Three articles satisfied the inclusion criteria. An additional three articles were yielded through a full-text review of nine articles retrieved through citation and website searching. A total of six studies were included in this narrative review (see Fig. 2). The individual study characteristics are described in Table 1. Publication dates spanned the years 2014 to 2022. Countries of publication included the USA [14–17], the UK [18], and Germany [19].

**Table 1:** Study characteristics

Authors robotic institution and year	Aims	Method	Participants	Results/outcomes
Culligan et al. Atlantic Health System (2014) [15]	To establish the predictive validity of a robotic surgery simulation training curriculum	Phase 1 Establishment of training protocol and expert benchmarks Phase 2 Online orientation; completion of simulator skills and da Vinci pig laboratory training Phase 3 Completion of robotic-assisted supracervical hysterectomy	5 gynecology surgeons (> 75 robotic cases per year) 14 OB/GYN board-certified study surgeons, with no prior robotic surgery training 4 control gynecology surgeons who were able to perform unsupervised robotic hysterectomies but had not been exposed to the da Vinci skills simulator	All 14 study surgeons completed the training protocol with an average of 20 h of simulator time Mean hysterectomy operative time = 20.2 min (experts); 21.7 min (study surgeons); 30.9 min (control) Mean expected blood loss = 25 ml (experts); 25.4 ml (study surgeons); 31.25 (control) Mean GOALS scores = 50 (experts); 34.7 (study surgeons); 31.1 (control)
Frederick et al. A.T.L.A.S Program, Roswell Park Cancer Institute (2016) [19]	To develop and validate a robotic hysterectomy assessment to objectively measure robotic surgical skills	Phase 1 Development of the assessment tool via expert Delphi consensus Phase 2 Evaluation of video recorded procedures by blinded expert reviewers using the assessment tool	Delphi panel n = 25 gynecologic oncology attendings (> 1500 robotic console hours) 20 senior gynecologic oncology fellows (100 to 400 robotic console hours) 7 novices	6 key domains pertaining to critical elements of a robotic hysterectomy were used to assess technical skills, with a maxim score of 80 Experts scored higher in all domains except vaginal cuff closure Advanced beginners scored higher than the novice group in all individual domains except performing colpotomy and vaginal cuff closure
Rusch et al. SERGS (2018) [16]	To test the feasibility of a pilot curriculum for robotic gynecological surgery and to verify its effectiveness in evaluating robotic surgical performance	Tri-modular course: in-house didactic introduction and virtual training; dry and wet laboratory training; in-house training and mentoring. Formal approval of a completed logbook and assessment of a video-case recording by a SERGS expert	4 fellows (surgical experience ranging from junior resident to staff surgeon), with little to no robotic surgical experience	All fellows performed a robotic hysterectomy without supervision and achieved good or acceptable technical quality General comment for more specified outline of the final curriculum as well as the evaluation and assessment process
Ismail et al. BIARGS (2020) [18]	To determine a list of competencies as the basis of a core robotic gynecology surgery curriculum for first assistant and console training	4 round Delphi study: Round 1: proposal of criteria/standards Round 2: scoring each of the standards using a five-point Likert scale Round 3: re-score considering the weighted mean scores of Round 2 Round 4: further round of scoring electronically	69 invitations to participate were sent to members and associates of the British and Irish Association of Robotic Gynaecological surgeons (BIARGS)	14 initial responses were received in Round 1, 16 responses in Round 2, 38 responses in Round 3 and 26 responses in Round 4 16 competencies were proposed for a first assistant, 26 for a console surgeon, and 4 standards for ongoing professional development Core skills for primary console surgeon consisted of 9 pre-surgery skills, 5 intraoperative but off-console skills and 12 on-console skills
Berges et al. John Hopkins University School of Medicine (2021) [14]	To evaluate the correlation between virtual reality simulation performance and intraoperative performance during a RALH	Participants completed 5 or 10 simulator exercises until they achieved a passing score, before the day of surgery and on the day of surgery. Participant’s intraoperative performance was then assessed by the attending surgeon using GEARS	12 PGY3, 7 PGY4 and 2 fellows (PGY5-7), 2 of which had prior robotic simulator experience/practice	There was a weak and negative correlation between overall intraoperative performance and overall simulation performance Advanced trainees (PGY4 and fellows) performed worse across all simulation exercises compared with PGY3 trainees with median scores of 78.8 and 86.8, respectively All trainees had comparable overall intraoperative performances, except for GEARS bimanual dexterity where more advanced trainees had a higher median score
Turner & Kim. University of Alabama at Birmingham (2021) [17]	To evaluate the feasibility of a simulation-based robotic hysterectomy curriculum and obtain objective measures of progress	Simulation sessions were held for each participant every 4 months for 1 year, progressing through the standardized steps of a robotic hysterectomy	31 OB/GYN residents (PGY1-4)	29/31 trainees successfully completed simulated hysterectomy in the allotted 30-min session by the end of the program Objective measurements universally improved with the range between PGY levels decreasing by session 4

SERGS Society of European Robotic Gynecological Surgery

BIARGS  British and Irish Association of Robotic Gynaecological Surgeons

RALH   Robotic-assisted laparoscopic hysterectomy

GEARS  Global Evaluative Assessment of Robotic Skills

### Development and identification of performance metrics

Individual details and specific metrics assessed by each study are represented in Table 2. Three studies identified both simulator-based and intraoperative metrics [14–16]. Two studies solely developed intraoperative metrics [18, 19], while Turner and Kim solely utilized simulator metrics.

**Table 2:** Description of metrics

Authors robotic institution and year	Simulator	Intraoperative	Measurement
Culligan et al. Atlantic Health System (2014) [15]	Exercises: peg board 2; matchboard 2; suture sponge board 2; tubes; ring walk 3; matchboard 3; camera targeting 2; energy dissection 1; energy dissection 2; energy switching 1 Metrics: drops; economy of motion; excessive instrument force; instrument collisions; instruments out of view; master workspace range; time to complete exercise	Operative time (time from first grasp of the uterine fundus to amputation of the uterus at the level of the cervical os); blood loss; GOALS score	Simulator-recorded parameters and GOALS score
Frederick et al. A.T.L.A.S Program, Roswell Park Cancer Institute (2016) [19]	x	Domain 1 (round ligament): identification and tension; incision with hemostasis Domain 2 (bladder flap): plane development; hemostasis; tissue handling of bladder Domain 3 (IP ligament): IP isolated; angle with device to secure pedicle; hemostasis Domain 4 (uterine vessels): isolation of uterine vessels; hemostasis Domain 5 (colpotomy): proper plane; hemostasis; exposure Domain 6 (vaginal cuff): suture placement; hemostasis; tissue approximation Total score; operative time	Robotic Hysterectomy Assessment Score (RHAS)
Rusch et al. SERGS (2018) [16]	Exercises: endo-wrist manipulation; energy and dissection; needle driving Metrics: total simulation score	Depth perception; bimanual dexterity; efficiency; force sensitivity; robotic control	GEARS/OSATS NOTSS
Ismail et al. BIARGS (2020) [18]	x	Core intraoperative skills divided into first assistant, console surgeon and commitment to continued surgical development Core skills for primary console surgeon consisted of 9 pre-surgery skills, 5 intraoperative but off-console skills and 12 on-console skills	x
Berges et al. John Hopkins University School of Medicine (2021) [14]	Exercises: energy dissection 1, camera targeting 1, match board 1, suture sponge 1 and tubes Metrics: time to complete; economy of motion; instrument collisions; instruments out of view; master workspace range; excessive instrument force; total simulation score	Depth perception; bimanual dexterity; efficiency; force sensitivity; robotic control; autonomy	Simulation score GEARS
Turner & Kim. University of Alabama at Birmingham (2021) [17]	Efficiency: total time; number of movements of left and right instruments; total path length of left and right instruments; total distance by camera; number of instrument collisions; number of clutches/clutch usage; total path/number of times instruments are out of view of camera; total time of instruments out of view of camera Safety: injury to bladder, colon, ureter, uterine artery and control, infundibulopelvic ligament and control, utero-ovarian ligament and control, large vessels of the pelvis	x	Simulation measured scores

Turner and Kim assessed trainee progress through their own curriculum entailing individual sessions on the simulator, whereas Rusch et al. based their training protocol on the validated curriculum used by European Association of Urology to teach robot-assisted urologic procedures [20]. Both studies identified objective metrics generated from their da Vinci robotic platform. Berges et al. identified specific simulator exercises most relevant to performing a robotic hysterectomy using a panel of expert surgeons and used simulator generated metrics to assess performance. Similarly, Culligan et al. obtained expert consensus to select high-value simulator skills from the robotic platform to then establish expert benchmarks for all parameters of these simulated skills.

Intraoperative metrics varied between the studies. Culligan et al. assessed performance through objective metrics including operative time, blood loss, and GOALS score. Similarly, both Rusch et al. and Berges et al. provided structured assessment through GEARS-associated metrics. Frederick et al. and Ismail et al. used a modified Delphi process to identify and describe objective metrics and desired competencies for gynecological robotic training. Using a panel of experts, the Delphi process established a level of consensus to create a set of standards and metrics. Ismail et al. developed competencies across three levels of expertise: medical first assistant, console surgeon, and continued professional development. Frederick et al. instead, obtained consensus to develop a scoring algorithm to be used in assessing robotic hysterectomy performance. This is the only example in the literature of a robotic hysterectomy-specific clinical skills assessment.

### Current status of validation of performance metrics in robotic hysterectomy

#### Content validity

Reported content validation measures for each study are shown in Table 3. Two studies reported that simulation skills on the da Vinci robot were validated through a panel of expert robotic surgeons [14, 15]. Culligan et al. further utilized the expert panel to develop benchmarks for all parameters of the surgical skills used to assess beginner performance. GEARS, a validated tool to assess robotic surgery performance [12], was used to define the intraoperative metrics in two articles [14, 16]. Two articles utilized a modified Delphi process, by obtaining consensus on each proposed metric. This occurred through multiple phases before they were included in the final referenced metrics [18, 19].

**Table 3:** Validity of metrics

Authors robotic institution and year	Reliability assessment	Content validation	Construct validation	Predictive validity
Culligan et al. Atlantic Health System (2014) [15]	Simulator-recorded measurements. Video recording of procedures assessed by two blinded observers using GOALS tool	Independent selection of skills by experts that were then compiled to create a list of 10 simulator skills to be used in the training protocol	Study surgeons significantly outperformed the control surgeons across all metrics except for mean GOALS score	Each study surgeon completed a live robotic hysterectomy. There was no significant difference between expert and study surgeons across all metrics except for GOALS score
Frederick et al. A.T.L.A.S Program, Roswell Park Cancer Institute (2016) [19]	CVI > 0.75 of Delphi panel	High-level consensus reached on the scoring system by Delphi panel	Experts outperformed both advanced beginners and novices in all individual domains except vaginal cuff closure	x
Rusch et al. SERGS (2018) [16]	Simulator recorded measurements. Video recording of procedures assessed by an external reviewer with final approval by the educational committee	GEARS domains previously validated	x	Each study surgeon completed a live robotic hysterectomy without supervision
Ismail et al. BIARGS (2020) [18]	IRR > 0.8 of Delphi panel	High-level consensus reached on the final standards by Delphi panel	x	x
Berges et al. John Hopkins University School of Medicine (2021) [14]	Simulator-recorded measurements. Intraoperative performance assessed immediately after the surgery by the attending surgeon	Identification of 5 simulator exercises most revelation to performing a robotic hysterectomy surgery, by a panel of expert surgeons	Higher level trainees did not perform better than novice trainees, with lower overall simulator scores	There was no correlation between total GEARS scores during RALH and simulator scores
Turner & Kim. University of Alabama at Birmingham (2021) (17)	Simulator-recorded measurements	x	Senior residents demonstrated higher scores compared to junior residents	x

### Construct validity

In this setting, construct validation refers to how useful a defined metric is in distinguishing between robotic surgical skill, i.e., determining skill level between novices to intermediates to experts. Of the articles that reported construct validity, three of four studies demonstrated that their metrics were able to distinguish between skill levels, although not all were statistically significant.

Culligan et al. compared outcomes between gynecology robotic surgery experts, novices, and control surgeons. At the completion of their training protocol, novices significantly outperformed the control surgeon group in all intraoperative metrics apart from blinded assessment of surgical skill using GOALS score [15]. Similarly, Frederick et al. demonstrated that the metrics included in their scoring tool achieved construct validity. By way of example, they showed that experts significantly outperformed both advanced beginners and beginners in all metrics except vaginal cuff closure, where advanced beginners did not perform significantly worse. The advanced beginner group scored significantly higher compared to beginners in all metrics except performing colpotomy [19]. The metrics described by Turner & Kim also demonstrated construct validity: senior residents performed better in all metrics compared to junior residents throughout their training program [17].

Interestingly, the robotic simulator metrics described by Berges et al. failed to achieve construct validity as advanced trainees performed worse across all simulator exercises compared to more junior trainees, although the differences were not significant [14]. However, all training levels achieved similar intraoperative GEARS scores suggesting that there is a limited correlation between robotic simulator performance and robotic hysterectomy performance. Indeed, there was an overall weak and negative correlation between intraoperative and simulation performance, suggesting that better intraoperative GEARS scores correlate with worse simulation performance.

### Predictive validity

Predictive validity in this context refers to the ability of a metric to predict future objective performance intraoperatively when performing a live robotic hysterectomy. Of the articles that reported intraoperative robotic hysterectomy performance, two demonstrated predictive validity. At the completion of a live robotic supracervical hysterectomy, Culligan et al. demonstrated that there was no significant difference between expert and trainee robotic surgeons across all metrics except for GOALS scores [15]. Rusch et al. also described improvement in GEARS score for fellows completing a robotic hysterectomy with or without adenectomy, achieving good or acceptable technical quality [16]. As discussed above, Berges et al. demonstrated limited correlation between simulator scores and total GEARS scores, suggesting that virtual reality simulator performance has limited correlation or validity in predicting intraoperative performance.

## Discussion

The challenges facing gynecological surgery training have been well documented [21–23]. Given the challenges of insufficient exposure and practice to obtain proficiency for novel robotic techniques, objective metrics of skill are required. Determining standardized and validated performance metrics is imperative for the development of safe and competent trainees [24]. In the absence of standardized training, this review presents the findings of current performance metrics that demonstrate promising validity in determining future intraoperative success.

### Determining metrics

The articles identified in this review demonstrated the use of expert consensus at multiple levels of the curriculum process. Three articles employed the widely used Delphi process to initially obtain specialist consensus on the perceived core components of robotic hysterectomy training and assessment programs [16, 17, 19]. In contrast, one article utilized experienced surgeons to establish expert benchmarks on specific robotic surgery simulation modules [18]. The Delphi process has been used to gauge and obtain an aggregate expert opinion on a particular topic; however, its effectiveness will depend on the availability of dedicated experts [19]. In summary, while Frederick et al. focused on intraoperative assessment using a procedure-specific tool, Rusch et al. addressed a broader system-level standardization of training. Nevertheless, both emphasize structured, validated assessments in robotic surgical training, straying from the conventional volume-based training requirements. This highlights the competency-based progression as an essential component of robotic hysterectomy training.

### Identifying metrics

As previously described, identifying metrics that are procedure-specific and objectively assesses performance level allows for improved surgical technique through the application of tailored feedback [24, 25]. Given the heterogeneous identification and development of performance metrics demonstrated in our studies, it is important to ascertain which method provides the most objective and valid metrics.

The metrics identified in this review could mostly be divided into robotic simulator-derived metrics and intraoperative metrics. In articles that included virtual simulator training [14–17], similar exercises and performance metrics were described, with the addition of safety metrics in one study [17]. Surgical simulation has been an important component of gynecologic surgery training for over a decade, designed to evaluate and provide trainees feedback on their surgical skills and improve the learning curve of robotic-assisted surgeries [26]. The limitations of surgical simulation have been well documented, with limited evidence in their utility to predict clinical outcomes. It has been suggested that an assessment of intraoperative performance by an attending surgeon may provide more holistic and haptic feedback that the simulator cannot capture [14, 21]. Indeed, it was demonstrated by Berges et al. that higher level trainees with more laparoscopic experience had worse total simulation scores.

Despite its wide use in gynecology training, many VR simulations have an uncertain correlation to hysterectomy surgery. Therefore, it is crucial to determine which simulator exercises are most relevant and yield the highest intraoperative outcomes. In Berges et al. and Culligan et al., both studies developed similar consensus-driven methods in which experts identified the exercises most relevant and helpful in performing a RALH. Exercises common to both studies included matchboard, camera targeting, energy dissection, and suture sponge [14, 15]. The selection of these exercises is further supported by previous studies that have validated its correlation with surgical training level as well as demonstrated that practice on these simulator skills improves intraoperative performance [27, 28]. Common simulator metrics identified by the studies included economy motion, instrument collisions and force, master workspace range, time to complete exercise and total score [14, 15, 17]. The da Vinci simulator was able to generate a performance score using these metrics ranging from 0 (worst performance) to 100 (best performance), providing an indication of trainee skill level.

The intraoperative metrics identified in the studies could further be divided into those that are procedure-specific and those that are generalizable to any operative procedure. Metrics that were generalizable included GEARS score domains such as depth perception, bimanual dexterity, efficiency, force sensitivity, and robotic control, as well as intraoperative time and blood loss [14–16]. In conjunction with total score and operative time, Frederick et al. described procedure-specific domains assessing trainee performance in each component of the RALH operation. Similarly, Ismail et al. described a total of 26 performance metrics encompassing the operative responsibilities of the first console surgeon, specific to RALH surgery. From these studies, it seems that using a modified Delphi methodology is the gold standard for ensuring content validity of performance metrics. Using the Delphi methodology, a consensus between experts can generate metrics that are objective and reflect the skill level required for the procedure. Given the aims of achieving competency-based training in robotic gynecology, it would be ideal to utilize an expert consensus-based methodology to identify and incorporate both generalized and procedure-specific metrics when assessing intraoperative performance.

### Construct and predictive validity

Our included studies called into question the ability of certain established metrics in distinguishing between surgical skill level accurately and objectively as well as predict in vivo intraoperative performance. Construct validity for GEARS has been demonstrated in previous studies, with one study concluding that GEARS was able to distinguish between skill levels in all parameters except depth perception [29, 30]. However, construct validity has not been measured specifically within the context of robotic hysterectomy.

Berges et al. and Culligan et al. both failed to achieve complete construct and predictive validity as their experts did not outperform novices in all domains. Berges et al. found no correlation between simulator scores and GEARS scores, with higher level trainees scoring on average lower overall simulator scores compared to novice trainees [14]. Similarly, experts outperformed novices in all metrics except for GOALS scores in Culligan et al.’s study [15]. Alongside, GEARS scores, Objective Structured Assessment of Technical Skill (OSATS) as well as Non-Technical Skills for Surgeons (NOTSS) were outlined for use in Rusch et al.’s curriculum. However, this form of feedback was not used as a standardized form of feedback by all supervisors, and thus their validity could not be assessed within the context of robotic hysterectomy surgery [16].

In this review, it was highlighted that GEARS has poor correlation between simulation scores and weak discrimination between surgical skills levels. Taken into consideration together, this information underscores the need for a more granular and assessment tool that not only differentiates between surgical skills level but also provides a more accurate representation of intraoperative performance. A possible method is the use of the Robotic Hysterectomy Assessment Score, as developed by Frederick et al. This robotic hysterectomy-specific scoring tool assesses the performance of key steps in a RALH on an anchored Likert scale, to provide tailored feedback on specific steps of the surgery [19]. Although further study is necessary to determine its efficacy in comparison to GEARS and other assessment tools. Another consideration is the use of a novel assessment method, e.g., cross-method validity, to select the most useful and reliable assessment tools when constructing a training curriculum [31]. Due to the lack of standardized assessment methods, novel training tools are unable to be assessed against ‘gold-standard’ metrics. As proposed by Hung et al., the cross-method validity process can correlate performance across different training methods (e.g., in vitro exercises, simulator performance and intraoperative performance) to determine the utility of novel performance metrics.

This work was undertaken to inform the design and integration of clinically relevant performance metrics into the IMRA robotic hysterectomy curriculum, which is a structured curriculum pathway for multiplatform hydrogel model-based training. This review has highlighted the gaps in the literature with a clear need for structured metric-based curricular development.

### Limitations

The scope of our review may have been limited by the narrow inclusion criteria and the limitation of English language studies only. A grey literature search strategy was not performed. A further possible limitation of our review is the assessment of metrics that were only referenced and validated within the context of robotic hysterectomy, which may narrow the scope of possible metrics that could be relevant and applicable to developing a robotic hysterectomy training program. Several studies were limited by their small cohorts and number of participants, potentially reducing the reliability of results. Furthermore, many of our included studies did not examine construct or predictive validity for their proposed metrics. Therefore, the metric’s translational capacity into intraoperative performance and success could not be completely assessed. For those that did address predictive validity, they did not address the translation into clinical outcomes.

## Conclusion

The aims of this review were to identify metrics of competence in robotic hysterectomy and to assess their validity in surgical training. This study highlights the lack of standardized performance metrics available to measure trainee skill level. The inconsistency in metric development and identification is reflected in the lack of validated assessment tools with the ability to differentiate between surgical skill level as well as provide an accurate indication of intraoperative performance. Together, this has culminated in a lack of standardized and validated robotic hysterectomy curriculums. This work informs ongoing work by IMRA to design and integrate clinically relevant performance metrics into robotic hysterectomy simulation curriculum design. 

## Data Availability

No datasets were generated or analysed during the current study.

## Appendix

See Figs. 1, 2

See Tables 1, 2, 3
